# Transcranial direct current stimulation (tDCS) in the treatment of neuropsychiatric symptoms of long COVID

**DOI:** 10.1038/s41598-024-52763-4

**Published:** 2024-01-25

**Authors:** Monika Klírová, Andrea Adamová, Nina Biačková, Olga Laskov, Veronika Renková, Zuzana Stuchlíková, Karolína Odnohová, Tomáš Novák

**Affiliations:** 1 National Institute of Mental Health, Topolová 748, 250 67 Klecany, Czech Republic; 2 Third Faculty of Medicine, Charles University, Prague, Czech Republic

**Keywords:** Outcomes research, Anxiety, Central nervous system infections

## Abstract

The study aimed to assess the efficacy of transcranial direct current stimulation (tDCS) in the treatment of neuropsychiatric (NP) symptoms of the post-acute sequelae of SARS-CoV-2 infection (PASC), known as the long COVID. A double-blind, randomized, sham-controlled study compared the efficacy and safety of prefrontal cortex active tDCS to sham-tDCS in treating NP-PASC. Patients diagnosed with NP-PASC, with a Fatigue Impact Scale (FIS) score ≥ 40, were eligible for the study. Twenty tDCS sessions were administered within four weeks, with continuous, end-of-treatment, and follow-up measurements. The primary outcome was a change in the FIS at the end-of-treatment, analyzed in the intention-to-treat population. Data from 33 patients assigned to active (n = 16) or sham-tDCS (n = 17) were analyzed. After the treatment, a decrease in the FIS score was more pronounced in the sham than in the active group, yet the intergroup difference was insignificant (11.7 [95% CI −11.1 to 34.5], p = 0.6). Furthermore, no significant intergroup differences were observed regarding anxiety, depression, quality of life, and cognitive performance. The small cohort sample, differences in baseline FIS scores between groups (non-stratified randomization), or chosen stimulation parameters may have influenced our findings. However, it might also be possible that the expected mechanism of action of tDCS is insufficient to treat these conditions.

## Introduction

The post-acute sequelae of SARS-CoV-2 infection (PASC), known as the post-COVID syndrome or the long COVID, is a set of distinct symptoms persisting more than one month after COVID-19^1^. Neuropsychiatric (NP) and neurocognitive symptoms are among the most common manifestations of PASC^1,2^. These include functional (such as fatigue (37%), sleep disturbances (31%), muscle pain (18%) or loss of sense of smell (12%)] and cognitive (such as brain fog (32%), memory (27%) or attention deficit (22%)) impairment, and emotional dysregulation (such as anxiety (23%) or depression (12%))^3^. They can vary from mild to severe symptoms, affecting the patient's daily life for several months^4^. Thus, PASC can represent a severe limitation that leads to reduced work capacity and quality of life and creates a critical need for therapeutic intervention.

Current therapeutic strategies for managing SARS-CoV-2-induced neuroinfection mainly involve pharmacological approaches with unclear results^5^. Given their limited efficacy in many patients, it is relevant to seek alternative therapeutic approaches. These could be based on influencing the pathophysiological mechanism of PASC, probably associated with persistent microvascular endotheliopathy, autoantibodies, localized inflammation, or reactivation of latent pathogens^6^. Transcranial direct current stimulation (tDCS), together with some other treatment strategies outlined in the consensus guidelines of the multidisciplinary collaboration^7^, might represent one such targeted treatment option for PASC treatment.

TDCS represents a Non-Invasive Brain Stimulation (NIBS) method, safe and user-friendly (portable and home-use)^8^ that has been proposed as an effective treatment tool for functional (e.g., fatigue)^9^, cognitive (e.g., attention or working memory impairment) impairment^10,11^, and emotional dysregulation (e.g., depression)^12–14^. In addition, tDCS has also been introduced as a treatment for fatigue^15^ in neuroimmune-based diseases (such as multiple sclerosis or post-polio syndrome) for its potential effect on restoring autonomic balance^16,17^. Current evidence suggests that tDCS can indirectly ameliorate inflammation manifestations by intervening in neuroplasticity processes^18^ or directly affecting NP symptoms persisting after infection^19^, raising the possibility of influencing the systemic immune response by targeting the frontal region^20,21^. Thus, tDCS might represent a potential therapeutic modality for managing the sequelae of COVID-19 infection not only as an adjunctive treatment to improve cognitive and physical rehabilitation^22^ but also to manage persistent symptoms after illness, such as fatigue or pain^19^.

Recently, the evidence for the effect of tDCS in the treatment of COVID-19 was mainly based on case reports^8,23^, but the results of several RCTs^24–31^ are now available. The first clinical RCTs in tDCS management of NP symptoms of acute and chronic stages of COVID-19 have confirmed its effect on influencing functional impairment, specifically alleviating fatigue^24–27^, cognitive impairment^28^, olfactory impairment^29^, and anxiety^25^. In addition, tDCS impacts other PASC symptoms, such as dyspnea^30^, and electrophysiological improvements^31^, such as heart rate variability or oxygen saturation. Those RCTs and case reports aimed at treating NP symptoms after COVID-19 targeted mainly the prefrontal (PFC) areas, such as the left dorsolateral^24^, the medial PFC^8,31–33^, or the primary motor cortex^25–27^, and found several positive results on fatigue^24–27^, anxiety^8,23,25,32,33^, and depression^8,23,33,34^ alleviation, and cognitive performance^8,25,28,29,33–36^ and quality of life^25^ improvement. However, research in this area is limited, and the evidence needs to be supported by the results of further studies.

Here, we investigated the effect of PFC-tDCS on fatigue alleviation in PASC. To this end, in a double-blind, parallel-group, sham-controlled study in patients with NP symptoms of PASC presenting chronic fatigue, we administered a 4-week tDCS procedure. We hypothesized that active tDCS would induce significantly higher fatigue relief than sham tDCS. As a secondary objective, we examined the effects of PFC-tDCS on changes in affective and anxiety symptoms, cognition, and quality of life.

## Results

**Figure 1:**
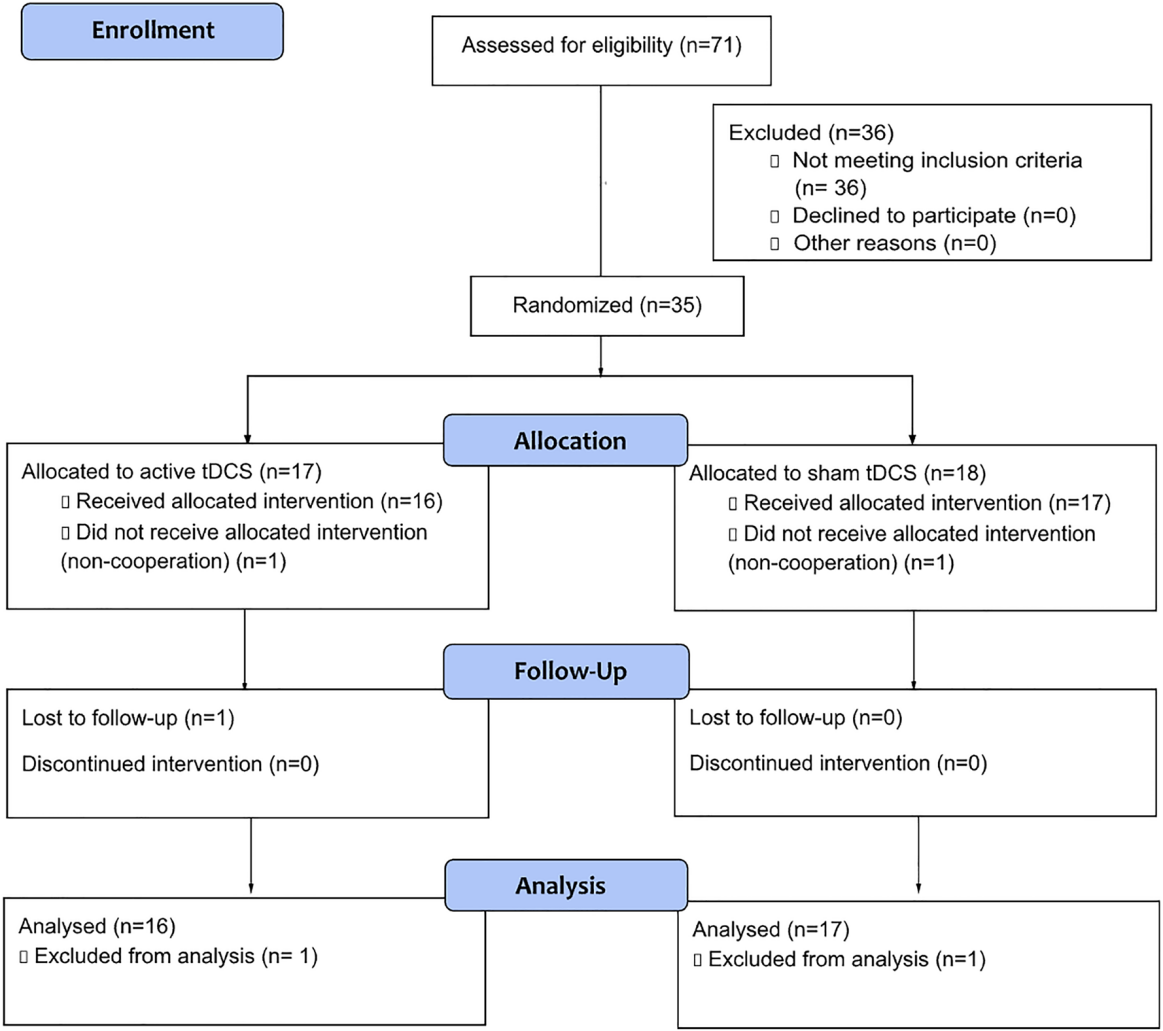
Consort study flow diagram.

Seventy-one patients were screened for eligibility between April 2022 and May 2023, and 35 met the inclusion criteria and consented to participate. One patient from each group withdrew from the study before the initial treatment. The final sample for analyses consists of thirty-three patients (16 in the active and 17 in the sham groups) who entered the blinded phase, and all consequently completed the four-week study. Within the one-month post-study observation, one participant from the active group was lost to follow-up (Fig. 1).

Twenty-three of thirty-three participants were women (70%), and the mean age was 42.2 ± 10.5 years. The duration of PASC was 13.3 ± 7.6 months. Eleven participants (33%) had a history of mental disorders, primarily anxiety disorders. Fifteen participants (46%) were taking antidepressants, and four (12%) were using benzodiazepines (BZDs) or anticonvulsants (AC) at the time of study entry.

The baseline characteristics of groups were comparable across various demographic and clinical parameters; however, except for a significant difference in the fatigue severity, patients in the sham group reported significantly higher scores in the Fatigue Impact Scale (FIS) total and cognitive and psychosocial domains (Table 1).

**Table 1:** Baseline demographic and clinical characteristics.

	Active (n = 16)	Sham (n = 17)	p-value
Age	44.4 (10.7)	40.1 (10.2)	0.25
Sex: F	11 (69)	12 (71)	1.00
BMI	26.9 (6.2)	25.9 (6.9)	0.46
Months since COVID-19	12.2 (8.3)	14.4 (7.0)	0.49
The severity of COVID-19: mild/moderate/severe	5/8/2002	5/9/2002	0.99
Hypertension	4 (25)	4 (23.5)	1.00
Diabetes mellitus type 2	2 (12.5)	1 (5.8)	0.60
Dyslipidemia	0 (0)	3 (17.6)	0.23
Hypothyroidism	3 (18.7)	5 (29.4)	0.69
History of psychiatric illness	4 (25)	7 (41)	0.47
Current use of psychiatric drugs	7 (44)	11 (65)	0.30
Antidepressants	5 (31)	10 (59)	0.17
BZD/AC	1 (6)	3 (18)	0.60
GAD-7	10.7 (5.6)	10.3 (5.3)	0.84
PHQ-9	12.1 (5.3)	13.9 (3.6)	0.25
CGI	4.0 (0.6)	4.1 (0.9)	0.98
FIS			
FIS total	77.2 (28.5)	101.6 (25.2)	**0.01**
FIS cognitive	21.1 (8.6)	28.6 (6.2)	**0.01**
FIS physical	20.4 (7.8)	23.9 (8.6)	0.22
FIS psychosocial	35.7 (14.9)	49.1 (15.0)	**0.02**
A-PASC			
A-PASC total	71.1 (21.9)	83.4 (22.5)	0.12
A-PASC physical	26.1 (12.6)	32.6 (13.1)	0.16
A-PASC cognitive	19.1 (6.8)	21.3 (5.7)	0.32
A-PASC emotional	7.0 (4.4)	7.2 (4.2)	0.91
A-PASC functional	18.6 (5.2)	22.3 (5.7)	0.06
AQoL-6D			
AQoL total	61.4 (11.3)	55.3 (13.1)	0.16
AQoL senses	81.7 (10.8)	70.1 (11.2)	**0.01**
AQoL pain	60.8 (28.2)	53.5 (30.6)	0.48
AQoL independent living	69.4 (12.3)	62.7 (17.5)	0.21
AQoL relationships	66.3 (16.7)	58.2 (16.7)	0.18
AQoL mental health	42.6 (20.7)	45.2 (20.1)	0.71
AQoL managing	49.0 (18.5)	40.7 (12.8)	0.15
Digit span			
fTE_ML	5.9 (2.7)	6.1 (1.4)	0.88
fML	6.8 (2.2)	6.6 (1.9)	0.81
bTE_ML	5.3 (2.6)	4.9 (1.8)	0.64
bML	5.8 (2.4)	5.8 (1.7)	0.99
Digit symbol substitution test			
Correct count	44.5 (15.9)	41.4 (21.7)	0.64

Anticonvulsants (AC); Post-COVID-19 Symptoms Assessment Questionnaire (A-PASC); Assessment of Quality of life—six dimensions (AQoL-6D); Body Mass Index (BMI); Maximal backward digit span that a participant recalled correctly during all 14 trials. It is set to 0 before the start of the Digit Span (bML); Two-error maximum length, the traditional measure of a participant's backward digit span. It is the last digit span a participant gets correct before making two consecutive errors (bTE_ML); Benzodiazepines (BZD); Clinical Global Impression (CGI); Female (F); Fatigue Impact Scale (FIS); Maximal forward digit span that a participant recalled correctly during all 14 trials (fML); Two-error maximum length, the traditional measure of a participant's forward digit span. It is the last digit span a participant gets correct before making two consecutive errors (fTE_ML); Generalised Anxiety Disorder (GAD-7); Patient Health Questionnaire (PHQ-9).

Data are presented as mean (SD) or number of cases (%). Comparisons between groups were assessed using unpaired t-test, Mann–Whitney U test, or Fisher exact test as appropriate.

Significant values are in bold.

**Figure 2:**
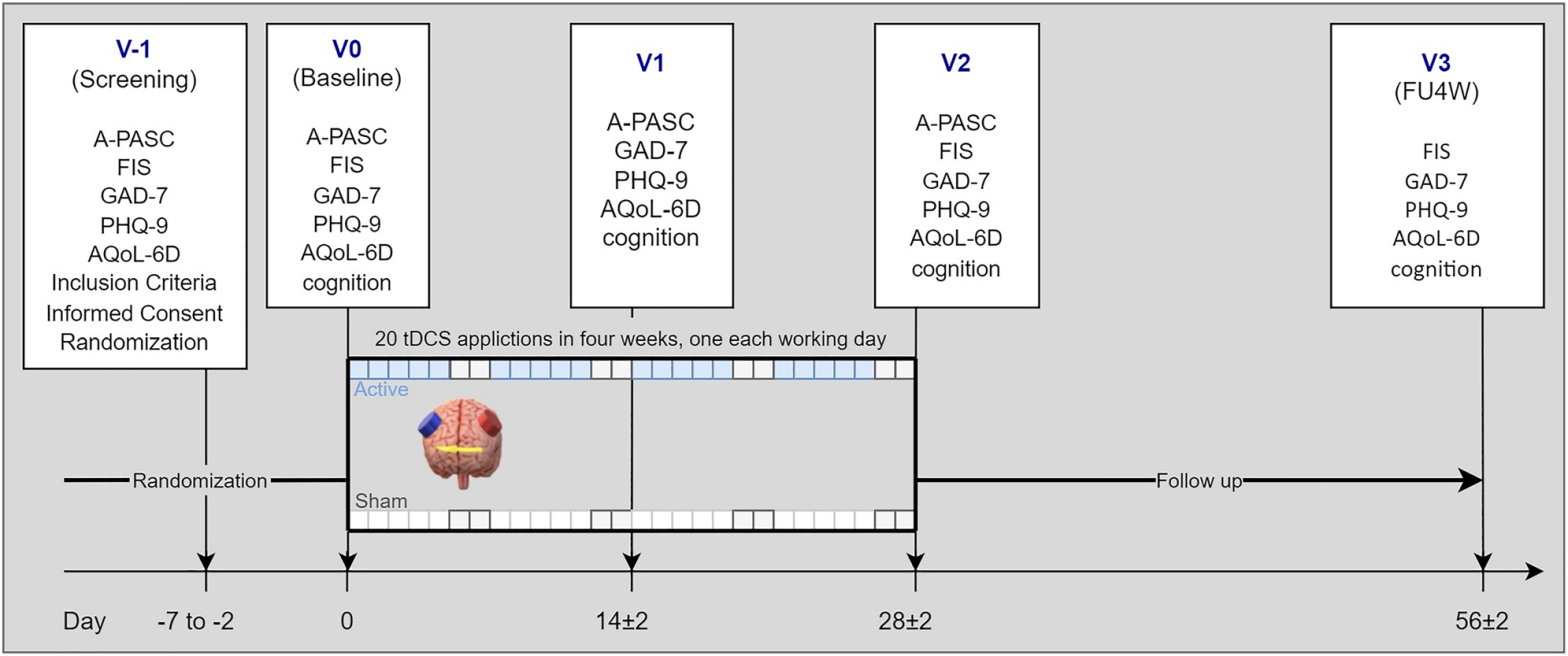
Scheme of the study. Post-COVID-19 Symptoms Assessment Questionnaire (A-PASC); Assessment of Quality of Life—six dimensions (AQoL-6D); Clinical Global Impression (CGI); Fatigue Impact Scale (FIS); Generalised Anxiety Disorder (GAD-7); Patient Health Questionnaire (PHQ-9); Transcranial Direct Current Stimulation (tDCS).

**Figure 3:**
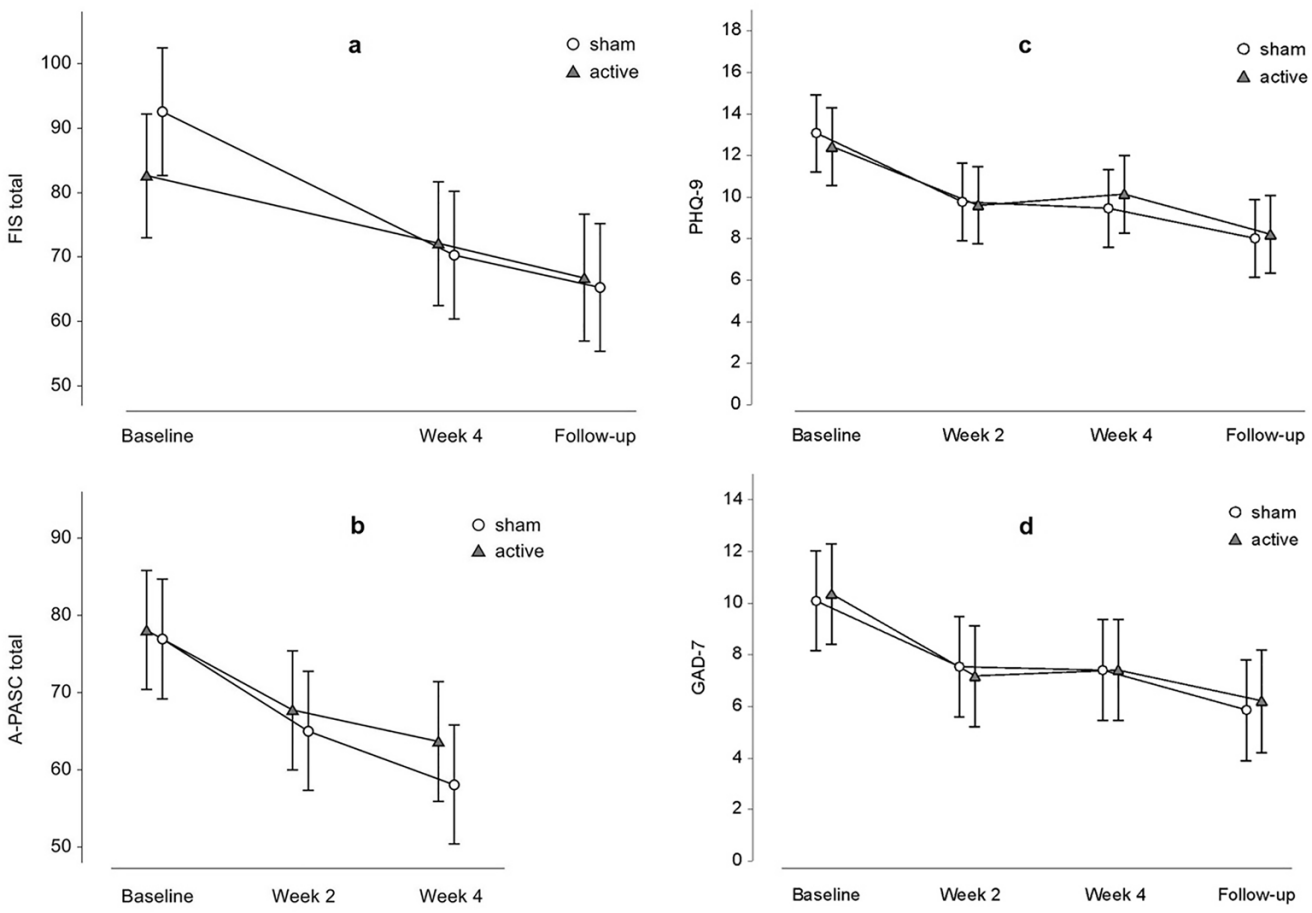
Clinical outcomes. (**a**) Change in Fatigue Impact Scale (FIS); (**b**) change in Post-COVID-19 Symptoms Assessment Questionnaire (A-PASC); (**c**) change in self-assessment of depression using Patient Health Questionnaire (PHQ-9); (**d**) change in self-assessment of anxiety using Generalised Anxiety Disorder (GAD-7). Symbols represent least-squares means and error bars their 95% confidence intervals.

At the end of the four-week randomized control trial (Fig. 2), there was a substantial decrease in fatigue severity according to the FIS scale, but no difference between groups was revealed (the mixed model for repeated measures (MMRM); effect of time: F(2,55.6) = 11.88, p < 0.001; effect of treatment: F (1,23.6) = 0.10, p = 0.8; treatment x time interaction: F (2,53.3) = 1.07, p = 0.35). The least squares (LS) mean difference between groups in the FIS total score changes from the baseline to week 4 (the primary outcome endpoint) was 11.7 points (95% CI −11.1 to 34.5, t = 1.36, p_corr_ = 0.6). Within-group comparisons showed significant improvement only in the sham group (sham: −22.2, 95% CI −40.3 to −4.1), t = 3.6, p_corr_ = 0.005; active: −10.5, 95% CI −28.0 to 7.0, t = 1.76, p_corr_ = 0.7). Continuous alleviation of fatigues was also observed at the end of follow-up, however still nonsignificant in the active group (active vs sham: 11.3 (95% CI −11.7 to 34.4), t = 1.31, p_corr_ = 0.7; sham: −27.1 (95% CI −45.2 to −9.1), t = 4.40, p_corr_ < 0.001; active: −15.8 (95%CI −33.7 to 2.1), t = 2.59, p_corr_ = 0.13) (Fig. 3a). Improvement of fatigue from baseline to end of the study and to follow-up were also found for cognitive, physical, and psychosocial domains; again, neither achieved significant between-group differences (Table 2).

**Table 2:** PASC severity change over the study period.

tDCS		Active (n = 16)			Sham (n = 17)			Time × condition					
		Baseline (V0)—during tDCS (V1)	Baseline (V0) post-tDCS (V2)	Baseline (V0)—FU-4W (V3)	Baseline (V0)—during tDCS (V1)	Baseline (V0) post-tDCS (V2)	Baseline (V0)—FU-4W (V3)	Active vs. sham (V0–V1)		Active vs. sham (V0–V2)		Active vs. sham (V0–V3)	
		LS mean (95% CI)	LS mean (95% CI)	LS mean (95% CI)	LS mean (95% CI)	LS mean (95% CI)	LS mean (95% CI)	LS mean (95% CI)	P	LS mean (95% CI)	p	LS mean (95% CI)	p
FIS	Total	–	−10.50 (−28.00; 7.00)	−15.80 (−33.65; 2.05)	–	− 22.2 (−40.3; −4.1)	−27.13 (−45.21; −9.06)	–	–	11.7 (−11.14; 34.54)	0.626	11.33 (−11.74; 34.40)	0.666
	Cognitive	–	−2.75 (−8.41; 2.91)	−2.49 (−8.26; 3.28)	–	−7.80 (−13.64; −1.96)	−9.47 (−15.31; −3.62)	–	–	5.05 (−2.34; 12.44)	0.321	6.97 (−0.49; 14.43)	0.077
	Physical	–	−2.31 (−7.09; 2.46)	−4.58 (−9.38; 0.38)	–	−4.33 (−9.26; 0.60)	−4.93 (−9.86; −0.00)	–	–	2.02 (−4.21; 8.25)	0.917	0.43 (−5.87; 6.72)	1.000
	Psychosocial	–	−5.44 (−14.46; 3.58)	−8.57 (−17.78; 0.64)	–	−10.07 (−19.38; −0.75)	−12.73 (−22.05; −3.42)	–	–	4.63 (−7.14; 16.40)	0.832	4.17 (−7.73; 16.06)	0.889
A−PASC	Total	−10.38 (−22.04; 1.29)	−14.44 (−26.10; −2.77)	–	−9.60 (−21.65; 2.45)	−16.60 (−28.65; −4.55)	–	−0.78 (−16.00; 14.45)	1.000	2.16 (−13.06; 17.38)	0.998	–	–
	Physical	−4.69 (−10.68; 1.30)	−5.44 (−11.43; 0.55)	–	−3.27 (−9.46; 2.92)	−5.07 (−11.26; 1.12)	–	−1.42 (−9.24; 6.40)	0.993	−0.37 (−8.19; 7.45)	1.000	–	–
	Cognitive	−0.63 (−4.05; 2.80)	−3.69 (−7.12; −0.26)	–	−2.47 (−6.01; 1.07)	−5.53 (−9.07; −1.99)	–	1.84 (−2.63; 6.31)	0.805	1.85 (−2.63; 6.32)	0.803	–	–
	Emotional	−2.00 (−4.49; 0.49)	−1.56 (−4.06; 0.93)	–	−1.73 (−4.31; 0.84)	−2.07 (−4.64; 0.51)	–	−0.27 (−3.51; 2.99)	1.000	0.50 (−2.75; 3.76)	0.997	–	–
	Functional	−2.63 (−6.42; 1.17)	−3.31 (−7.11; 0.49)	–	−2.13 (−6.06; 1.79)	−3.93 (−7.86; −0.01)	–	−0.49 (−5.45; 4.46)	1.000	0.62 (−4.34; 5.58)	0.999	–	–
GAD 7		−3.19 (−6.64; 0.26)	−2.94 (−6.39; 0.51)	−3.92 (−7.44; −0.41)	−2.56 (−6.01; 0.89)	−2.69 (−6.14; 0.76)	−4.25 (−7.70; −0.80)	−0.63 (−4.92; 3.67)	1.000	−0.25 (−4.55; 4.05)	1.000	0.33 (−4.02; 4.67)	1.000
PHQ 9		−2.81 (−6.16; 0.53)	−2.25 (−5.59; 1.09)	−4.19 (−7.53; −0.84)	−3.31 (−6.66; 0.03)	−3.63 (−6.97; −0.28)	−5.06 (−8.41; −1.72)	0.50 (−3.67; 4.67)	1.000	1.38 (−2.79; 5.54)	0.959	0.88 (−3.29; 5.04)	0.997
AQoL−6D	Total	4.14 (−3.21; 11.49)	3.83 (−3.52; 11.18)	7.66 (0.30; 15.01)	8.31 (1.18; 15.44)	7.20 (0.07; 14.34)	10.88 (3.75; 18.02)	−4.17 (−13.19; 4.85)	0.805	−3.38 (−12.40; 5.64)	0.923	−3.23 (−12.25; 5.79)	0.939
	Senses	−0.96 (−6.75; 4.83)	0.001 (−5.79; 5.79)	0.0006 (−5.79; 5.79)	4.07 (−1.55; 9.69)	4.98 (−0.64; 10.60)	7.24 (1.62; 12.86)	−5.03 (−12.14; 2.07)	0.327	−4.98 (−12.08; 2.13)	0.341	−7.24 (−14.34; −0.13)	**0.043**
	Pain	3.93 (−10.31; 18.17)	3.93 (−10.31; 18.17)	5.63 (−8.61; 19.87)	4.81 (−9.00; 18.63)	0.54 (−13.28; 14.35)	4.28 (−9.53; 18.09)	−0.89 (−18.36; 16.58)	1.000	3.39 (−14.08; 20.86)	0.998	1.35 (−16.12; 18.82)	1.000
	Independent living	1.39 (−6.98; 9.76)	2.78 (−5.59; 11.15)	6.25 (−2.12; 14.62)	5.55 (−2.56; 13.67)	9.48 (1.36; 17.59)	10.78 (2.66; 18.90)	−4.17 (−14.43; 6.10)	0.888	−6.70 (−16.96; 3.57)	0.428	−4.53 (−14.80; 5.73)	0.838
	Relationships	1.88 (−8.90; 12.65)	−0.63 (−11.40; 10.15)	3.13 (−7.65; 13.90)	6.47 (−3.99; 16.93)	4.12 (−6.34; 14.57)	4.71 (−5.75; 15.16)	−4.60 (−17.82; 8.63)	0.947	−4.74 (−17.97; 8.48)	0.938	−1.58 (−14.80; 11.64)	1.000
	Mental health	12.50 (−0.64; 25.64)	12.11 (−1.03; 25.25)	18.75 (5.61; 31.89)	16.54 (3.80; 29.29)	10.66 (−2.08; 23.41)	19.12 (6.37; 31.86)	−4.04 (−20.16; 12.07)	0.991	1.45 (−14.67; 17.56)	1.000	−0.37 (−16.48; 15.75)	1.000
	Managing	4.69 (−5.94; 15.31)	2.08 (−8.54; 12.71)	8.85 (−1.77; 19.48)	10.78 (0.48; 21.09)	10.29 (−0.01; 20.60)	15.19 (4.89; 25.50)	−6.10 (−19.13; 6.94)	0.795	−8.21 (−21.24; 4.82)	0.472	−6.34 (−19.37; 6.69)	0.762
Digit span (forward)	fTE_ML	1.01 (−0.34; 2.36)	0.06 (−1.26; 1.38)	0.61 (−0.74; 1.96)	−0.18 (−1.46; 1.10)	0.65 (−0.66; 1.96)	0.81 (−0.53; 2.14)	1.18 (−0.46; 2.82)	0.306	−0.59 (−2.22; 1.05)	0.939	−0.20 (−1.87; 1.47)	1.000
	fML	0.24 (−0.70; 1.17)	0.19 (−0.73; 1.10)	0.44 (−0.50; 1.37)	0.12 (−0.77; 1.00)	0.60 (−0.31; 1.50)	0.76 (−0.17; 1.68)	0.12 (−1.02; 1.25)	1.000	−0.40 (−1.54; 0.73)	0.940	−0.32 (−1.48; 0.84)	0.985
Digit span (backwards)	bTE−ML	0.35 (−1.02; 1.73)	0.75 (−0.60; 2.10)	1.02 (−0.36; 2.40)	0.71 (−0.60; 2.01)	0.98 (−0.36; 2.31)	1.01 (−0.35; 2.38)	−0.35 (−2.03; 1.32)	0.997	−0.23 (−1.90; 1.44)	1.000	0.01 (−1.70; 1.72)	1.000
	bML	0.71 (−0.39; 1.83)	0.63 (−0.46; 1.71)	0.98 (−0.13; 2.10)	0.59 (−0.47; 1.64)	0.67 (−0.41; 1.75)	0.62 (−0.47; 1.73)	0.13 (−1.22; 1.48)	1.000	−0.04 (−1.39; 1.31)	1.000	0.36 (−1.02; 1.74)	0.989
Digit symbol substitution test (correct symbols)		4.59 (−5.27; 14.45)	5.88 (−3.54; 15.29)	6.88 (−2.75; 16.52)	13.69 (4.38; 23.01)	8.79 (−0.54; 18.12)	15.69 (6.15; 25.23)	−9.10 (−21.07; 2.86)	0.246	−2.92 (−14.61; 8.77)	0.992	−8.81 (−20.76; 3.15)	0.281

Post−COVID-19 Symptoms Assessment Questionnaire (A-PASC); Assessment of Quality of life—six dimensions (AQoL-6D); Maximal backward digit span that a participant recalled correctly during all 14 trials. It is set to 0 before the start of the Digit Span (bML); Two-error maximum length, the traditional measure of a participant's backward digit span. It is the last digit span a participant gets correct before making two consecutive errors (bTE_ML); Fatigue Impact Scale (FIS); Maximal forward digit span that a participant recalled correctly during all 14 trials (fML); Two-error maximum length, the traditional measure of a participant's forward digit span. It is the last digit span a participant gets correct before making two consecutive errors (fTE_ML); Generalized Anxiety Disorder (GAD-7); Patient Health Questionnaire (PHQ-9); Baseline visit (V0), Visit at a 2-week point during tDCS (V1), Post-tDCS visit (V2), 4-week follow-up visit (V 3 FU4W); Transcranial Direct Current Stimulation (tDCS).

Data show within-group least squares (LS) mean changes from baseline and between-group differences in LS means changes from baseline with 95% confidence intervals (Sidak-corrected).

In the more specific assessment for PASC, i.e., Post-COVID-19 Symptoms Assessment Questionnaire (A-PASC), the score decrease was revealed at week four compared to baseline (time: F(2,56.8) = 12.62, p < 0.001) with similar change within either group (active: −14.4 (95% CI −26.1 to −2.8), t = 3.62, p_corr_ = 0.004; sham: −16.6 (95% CI −26.7 to −4.6), t = 4.12, p_corr_ = 0.001), thus no between-group differences (treatment: F(1, 24.3) = 1.80, p = 0.19; treatment × time: F(2, 56.8) = 0.17, p = 0.8; active vs sham: 2.2 (95%CI −13.1 to 17.4), t = 0.30, p_corr_ > 0.9) (Fig. 3b). For subscales (cognitive, emotional, physical, and functional), analogous changes were found (Table 2). A-PASC was not administered at the follow-up visit.

Anxious and depressive symptoms were of mild to moderate severity at baseline and continued to decrease throughout the study and follow-up. Again, the decline in scores on the respective self-assessment questionnaires (Generalised Anxiety Disorder (GAD-7) and Patient Health Questionnaire (PHQ-9) was comparable between groups at all post-baseline visits (Fig. 3c,d, Table 2).

**Figure 4:**
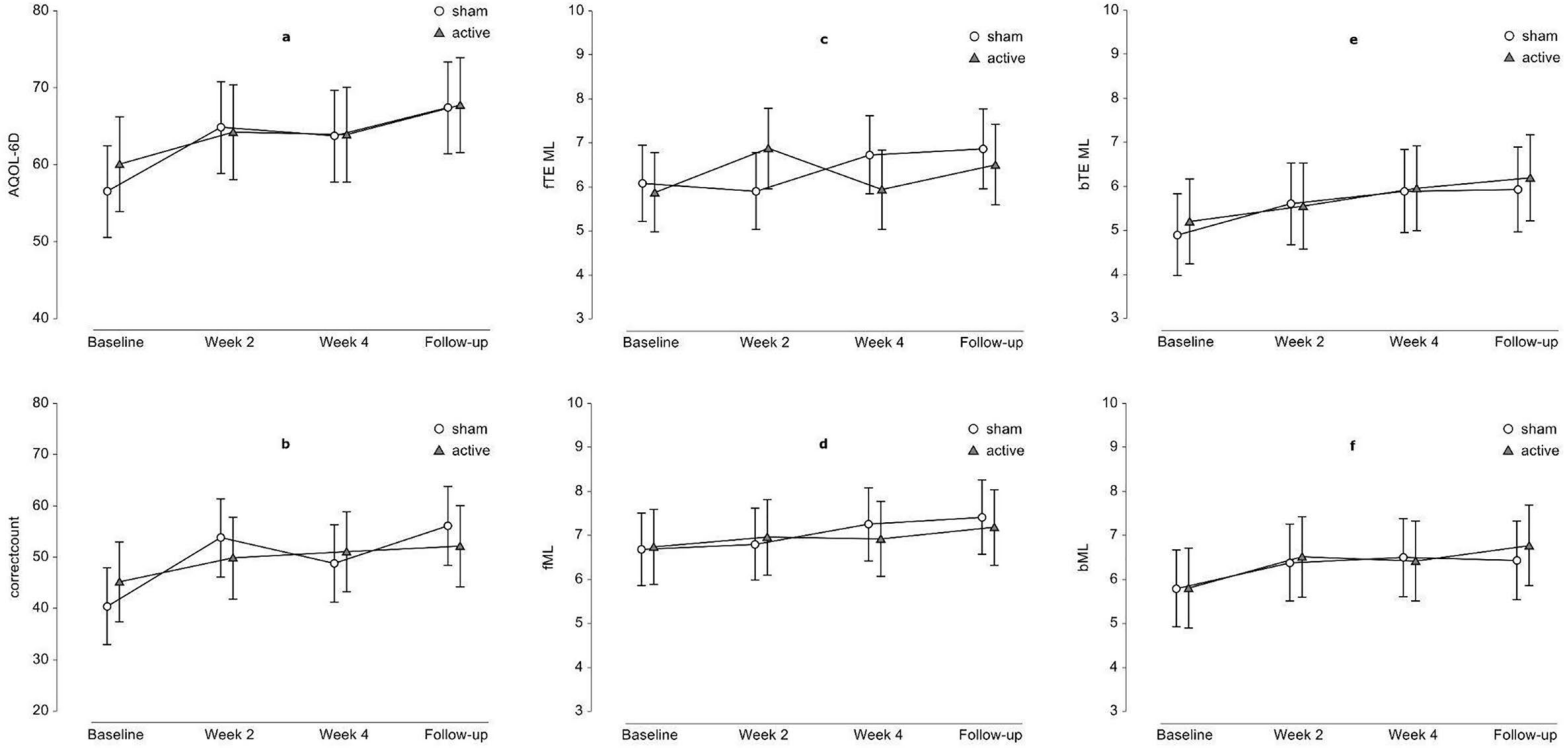
Quality of life and cognition outcomes. (**a**) Assessment of Quality of life—six dimensions (AQoL-6D); (**b**) correct symbols in Digit Symbol Substitution Test (correctcount); (**c**) two-error maximum length, the traditional measure of a participant's forward digit span. It is the last digit span a participant gets correct before making two consecutive errors (fTE_ML); (**d**) the maximal forward digit span that a participant recalled correctly during all 14 trials (fML); (**e**) two-error maximum length, the traditional measure of a participant's backward digit span. It is the last digit span a participant gets correct before making two consecutive errors (bTE_ML); (**f**) maximal backward digit span that a participant recalled correctly during all 14 trials. It is set to 0 before the start of the Digit Span (bML); Symbols represent least-squares means and error bars their 95% confidence intervals.

There was no difference in the quality of life as assessed by the Assessment of Quality of life—six dimensions (AQoL-6D) between groups during the study and follow-up (Fig. 4a, Table 2), except in the dimension of senses in follow-up measurement (Table 2), which also significantly differed between groups at baseline in favor of the sham group (Table 1). Subjects' performance on cognitive tests assessed by Digit Span—Forward (DSF)—attention, Digit Span—Backward (DSB)—working memory, and Digit Symbol Substitution Test (DSST)—psychomotor tempo were comparable between groups at all visits after the baseline examination. (Fig. 4b–f, Table 2).

During the initial 2 weeks of the RCT, eight of the sixteen patients (50%) receiving active tDCS and nine of the seventeen (53%) patients assigned to the sham tDCS group experienced mild to moderate side effects such as burning or tingling. The incidence of side effects subsided as the study continued, with five patients in each group (31 and 29%) reporting side effects by the end of the trial. After the one-month post-study follow-up, none of the patients reported ongoing side effects, indicating their transient nature.

## Discussion

So far, only a few studies have been published on the effect of tDCS in NP-PASC involving fatigue. Our study evaluated the efficacy and safety of tDCS in treating NP-PASC. The primary outcome was the FIS (fatigue) change at the endpoint, analyzed in the intention-to-treat population. Secondary outcome measures were a change in the FIS at follow-up and changes in A-PASC (post-covid symptoms), GAD-7 (anxiety symptoms), PHQ-9 (depressive symptoms), AQoL-6D (quality of life), and the cognitive tests: DSF (attention)/DSB (working memory) and DSST (psychomotor tempo) at the endpoint and the follow-up measurement. No intergroup difference was found in the FIS change and changes of secondary outcomes (A-PASC, GAD-7, PHQ-9, AQoL-6D, cognitive tests) at the endpoint or follow-up between the active and sham groups, except the change in the dimension of senses in the AQoL-6D in follow-up measurement, which we consider to be an incidental finding. There was no intergroup difference in the frequency, type, and severity of side effects^37^ monitored during the tDCS application. Predisposing factors for PASC (hypertension, hypothyroidism, type 2 diabetes mellitus, dyslipidemia) did not affect treatment outcomes.

### Sample size

At the time of study design, we could not rely on any RCT with this clinical population to estimate the sample size needed for the expected outcome (difference between active and placebo tDCS). It would also be difficult to rely on existing clinical RCTs focusing on other causes of fatigue, such as multiple sclerosis^38^, which have also included small samples.

It is questionable whether the results of this study may have been influenced by the small sample size, which was, however, adequately chosen with respect to recruitment opportunities between March 2022 and March 2023 in Czechia. The justification for expanding the sample size would have been warranted if this study had shown at least a trend favoring the active intervention. However, the results indicated the opposite—a non-significant difference and even a trend toward improvement in the sham group. The Bayesian factor (BF10 = 0.163) for the primary outcome (FIS total score) suggests that the observed data are about 6.12 times more likely under the null hypothesis (no difference between groups or sham is better) compared to the alternative hypothesis (active is better than sham), providing moderate to strong evidence against the benefit of active tDCS. Therefore, we believe our data reflect true negative results rather than false negatives.

### PASC cohort

The heterogeneous nature of PASC, based on differences in the clinical symptomatology of the PASC cohort, may also cause our findings. However, our analyses did not confirm the impact of the intergroup difference in the degree of clinical manifestation in baseline PASC characteristics (except for differences in FIS score—total and cognitive) or PASC predisposing factors such as severity of COVID-19, duration of PASC, predisposing physical illnesses, and history of psychiatric illness.

According to current knowledge, PASC can be caused by different pathophysiological mechanisms such as direct cytopathic response^39^, contribution to coagulation and vasculature vasculature-related issues^6,40,41^, dysregulation of the immune response facilitating reactivation of latent infections^40^, or by various psychological mechanisms^41^. Even the different variants of SARS-CoV-2 may directly affect the central nervous system to varying degrees^42^. Our study did not investigate which SARS-CoV-2 variant in the enrolled patients was responsible for NP-PASC. Considering that the enrolled patients underwent COVID-19 between April 2020 and November 2022 and that different SARS-CoV-2 virus variants, specifically 20/A; 20/B; 20/C; 20/E; Alpha: 20/I; Beta: 20/H; Gamma: 20/J; Kappa: 21/B; Delta: 21/A, 21/E, 21/I, 21/J; Omicron: 21/K, 21/L, 22/A, 22/B, 22/C, 22/D, 22/E, 22/F (from https://covariants.org/) occurred in Czechia during this time, we can infer that the heterogeneity of the cause of NP-PASC in the sense of SARS-CoV-2 variant may also have influenced the outcome of our study.

### Area of neuromodulation

**Figure 5:**
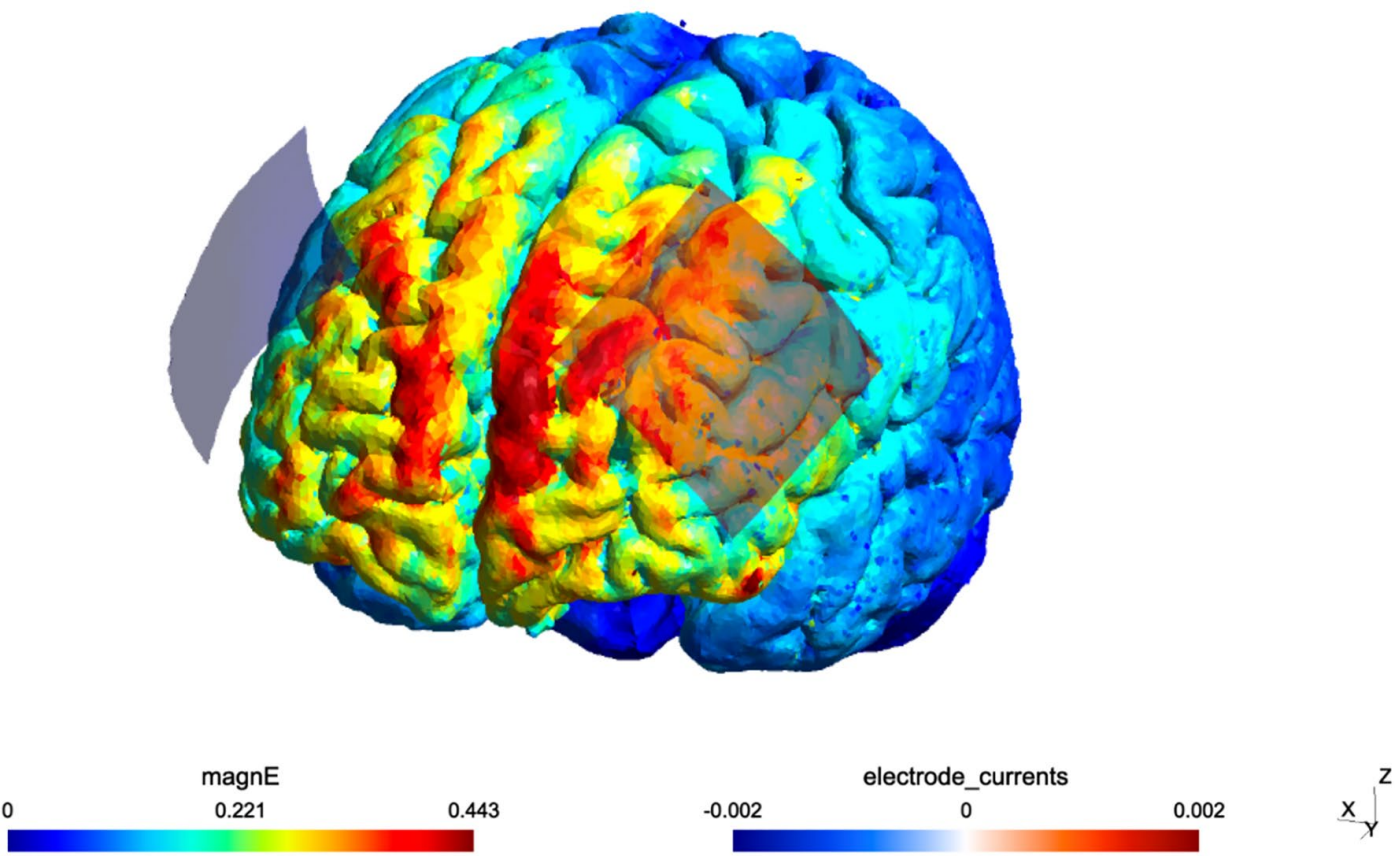
Visualization of the electric field simulation using SimNIBS 4.0 software. The computational modeling of the electric field was performed using SimNIBS 4.0 software; magnE is the magnitude of the electric field plotted at the gray matter surface measured in V/m.

The electrode placement (anode/cathode corresponding to the F3/F4 regions) for tDCS application was chosen based on the positive findings of one of the first published case reports in this area of research^8,23^. According to the visualization of the electric field simulation using SimNIBS 4.0 software, it corresponds to neuromodulation of the mPFC area (Fig. 5). In addition, this placement has been recently proven ineffective, for example, in treating depression^43^. It can be assumed that our choice of electrode placement may have accounted for the different outcomes of recent studies in PASC that have applied tDCS to other cortical regions, either to the left dorsolateral prefrontal cortex (DLPFC) or to the primary motor (M1) cortex. Specifically, left DLPFC tDCS showed relief from fatigue^24^ (change in the Modified FIS (MFIS) physical fatigue subdimension) and affected severity of depression^24^ (change in the Beck Depression Inventory (BDI)). Similarly, the tDCS of the M1 area alleviated fatigue (change in the MFIS or Fatigue Assessment/Severity Scale (FAS, and FSS)^25–27^, improved the subjective health (VAS-Health; EQ-5D-5L VAS]^27^, anxiety (Hamilton Anxiety Rating Scale)^25^, and quality of life (WHOQOL-BREF)^25^. Moreover, the M1 region seems suitable for tDCS application in other causes of fatigue, such as multiple sclerosis^15^ or stroke^44^.

### Stimulator parameter

Furthermore, the chosen stimulation parameters may have also influenced the outcome of this study.

#### Session duration

The results of our study may have been influenced by the duration of each tDCS session, which lasted 30 min. Thus, treatment protocols with different session duration (e.g., 20 min) may have yielded different results. It appears that the efficacy of tDCS does not necessarily increase with a longer duration of tDCS session^45,46^ and may even have the opposite effect^46^. Since some RCTs in PASC^24,26^ or other diseases causing fatigue^47–49^ have benefited from tDCS using a session duration of 20 min, it can be inferred that the chosen duration of the session and maybe even smaller number of sessions^15,47,50–52^ may have partially influenced our study results.

Intensity: We used a DC of 2 mA for the tDCS application. However, some existing studies on non-clinical populations suggest that excitatory after-effects are nonlinear with increasing intensity of tDCS^45,53^. Nevertheless, most NP clinical studies^13^, especially those focused on fatigue relief after tDCS, including PASC^24–27^ or multiple sclerosis^49^, have confirmed the beneficial effects of tDCS using a DC of 2 mA or higher intensity.

### tDCS as an augmentation of rehabilitation

PASC treatment typically involves a multidisciplinary approach, including intervention from different medical disciplines, with treatment plans for NP-PASC often focusing on psychopharmacological treatment of symptoms, such as antidepressants or physical or cognitive rehabilitation tailored to the individual patient's needs. In our case, tDCS was not used to augment an established rehabilitation procedure as another NP-PASC tDCS study did^25,28^. TDCS was applied during patient activities (reading, housework, etc.) that were individually difficult—no specific rehabilitation program was performed during tDCS. However, the choice of tDCS as an adjunct to targeted rehabilitation, as shown in some other studies^25,28,54^, may have led to different outcomes.

### Systemic factors and tDCS

Taking the possible reasons for our study findings more generally, it should be noted that tDCS has been investigated primarily for its potential to modulate neuronal activity^13^. Although recent studies have demonstrated an effect of this method on restoring autonomic balance^16,17^ or its indirect effect on manifestations of inflammation through neuroplasticity processes^18^, these mechanisms may be insufficient, especially when fatigue or other NP-PASC symptoms may be caused by systemic factors such as inflammation, immune dysregulation, or their combination. They may not directly address the complex underlying mechanisms that contribute to PASC.

### The main study limits

The main limitation of our study was the differences in baseline FIS scores between the active and sham groups. Patients assigned to the placebo group had a significantly higher FIS score at the baseline visit than those in the active group, which may have influenced the outcome. This could have been addressed by setting a higher FIS ceiling as an inclusion criterion or by stratified randomization at the time of study design.

Another shortcoming of the study is that, with the exception of cognitive tests, the reported results were obtained only from subjective questionnaires. The study lacks objective scales to assess clinical changes objectively.

Also, the study design did not include another active stimulation (e.g., with different electrode positions or with anode and cathode swapping) as a control group to verify the effect/non-effect demonstrated by tDCS.

The study was also limited by the severity of the participants' PASC—patients with severe symptoms would not be able to participate (inability to complete self-assessment questionnaires, self-operation of tDCS). In addition, most participants enrolled were searched through Facebook groups, indicating that this was a population that was able to actively seek information about PASC.

Another study flaw is the omission of a pre-treatment assessment of participants' expectations/perceptions of effects and the post-treatment recording of their guesses regarding their group allocation. Notably, higher expectations of a positive outcome may have contributed to the increased improvement in the placebo group (or the absence of a difference between groups). Indirectly, high expectancy may be inferred from the high retention rate in the study, as all participants who started the intervention completed the study.

## Conclusion

We did not find the active tDCS superior to sham tDCS in fatigue, anxiety, depression, and other PASC symptoms relief or quality of life and cognitive performance improvement in PASC. The small cohort sample, differences in FIS scores between groups at baseline, or chosen neuromodulation parameters, such as tDCS session duration or selection of mPFC instead of M1 cortex as a target area, may influence our findings. Also, it might be possible that the expected mechanism of action of tDCS is insufficient to treat these conditions.

## Patients and methods

### Subjects

Outpatients of the National Institute of Mental Health (NIMH) with a history of moderate or severe COVID-19 infection dispensed by an outpatient physician for neuropsychiatric problems (fatigue, anxiety, depressed mood, sleep disturbance, etc.) within the PASC were eligible for study participation. They were approached through advertising via Facebook groups, advertising on the NIMH website (https://www.nudz.cz), or by referral from their physician or psychiatrist. Recruitment for the study ran from March 2022 to March 2023. Before the study, patients' health status was assessed by a physician. Written informed consent was obtained from all subjects. The ethics committee of the NIMH approved the study (No. 46/23), and the study has been registered at ISRCTN with trial ID ISRCTN10942585. All methods used on the study subjects were performed in accordance with relevant guidelines and regulations.

Inclusion criteria were females or males aged 18 to 75 years; PCR RNA SARS-CoV-2 negativity at the time of screening/pre-study entry; symptom duration > 1 month after detection of COVID-19; FIS questionnaire score ≥ 40; the presence of neuropsychiatric symptoms of PASC as determined by the A-PASC questionnaire with a minimum total score ≥ 25; psychopharmacological medication (if used) on a stable dose for ≥ 4 weeks; competent to give informed written consent.

Patients were excluded if one of the following conditions was present: contraindications to tDCS (skin disease, superficial injury, and fracture or skull fracture in the area of stimulation, epilepsy, metal plates in the head); history of any other DSM-IV Axis I diagnosis prior to COVID-19, except a) depressive disorder, anxiety disorder, and sleep disorders or substance use disorder, which may be present in the history but with at least six months of documented symptom remission; pregnancy or breastfeeding; patients with severe or unstable somatic disorders (cardiovascular disease, neoplasms, endocrinological disorders, etc.); patients suffering from a neurological disorder (e.g. epilepsy, head injury with loss of consciousness).

### Procedure

The screening phase was conducted on days −2 to −7 before starting the active phase. After initial screening and study enrollment (see Clinical Assessment below), informed consent was obtained, and patients (n = 35) were randomized in a 1:1 ratio to one of two study groups: active DLPFC-tDCS and placebo-tDCS (block randomization). A baseline visit (day 0) was conducted on the day of tDCS initiation. Patients were subsequently clinically examined and assessed in the NIMH outpatient clinic at two weeks (day 14 ± 2) and at the end (day 28 ± 2) of four weeks of tDCS administration and after a four-week follow-up (FU-4W) visit (day 56 ± 2).

### Assessment of clinical status and cognition

A medical history (somatic status, presence of neuropsychiatric symptoms), including a detailed history of COVID-19 (course and duration of the disease, history of COVID-19 treatment, etc.), was taken at inclusion in the study.

Clinical assessment, including FIS assessment as the primary outcome at the end of the tDCS course, was performed using self-assessment scales (FIS; A-PASC; PHQ-9; GAD-7; AQOL-6D) and objective assessment (CGI) by the physician at each visit, except A-PASC (was not used in the FU-4W visit) and FIS (was not used in the visit at week two). Examination of cognitive functions (attention, working memory, and psychomotor tempo) was performed using computerized cognitive tests (Digit Span, DSST) with the assistance and instruction of a psychologist at each visit. TDCS tolerability was assessed using the tDCS self-assessment questionnaire^37^ after two weeks and at the end of treatment. The patients and the objective assessors were blinded to the patient group (active versus placebo-tDCS).

If the patient was taking psychopharmaceuticals (antidepressants, anxiolytics, hypnotics) for any reason (e.g., neurological indication, pain management, depression, or insomnia) at study entry, the patient was eligible to enter the study only if he/she had been taking the medication for at least four weeks before study entry and agree to continue taking it at an unchanged dose for the duration of study participation. The only additional psychopharmacological treatment for the patient's participation in the study was clonazepam (up to 1 mg daily) for anxiety and zolpidem (up to 10 mg daily) for insomnia for all groups.

### tDCS application

The HDCStim programmable stimulator (Newronica, Italy) was used for tDCS application in a double-blind design. The stimulating electrodes were placed as follows: the anode on the F3 area (the area for EEG electrode placement according to the international 10/20 system) and the cathode contralaterally on the F4 area. The electrodes with sponges filled with saline were fixed with the help of Mind-cap to the stimulating areas (F3—above the left DLPFC; F4—above the right DLPFC), which allows for possible home use (ensuring identical electrode placement during repeated application).

A total of 20 tDCS sessions (Monday to Friday) were administered to patients over a four-week period. During active tDCS, a DC of 2 mA intensity (current density 0.08 mA/m^2^) was applied for 30 min with an initial ramp-up and final ramp-down of current intensity, each lasting 30 s. Placebo-tDCS with an identical electrode assembly involved only an initial 30 s ramp-up phase, immediately afterward 30 s ramp-down phase to simulate sensations similar to active stimulation, then was stopped and followed by 29 min rest.

The tDCS applicator was blinded to the study participant's affiliation with the active or placebo group. For home use, the device was unlabelled, pre-programmed (active or placebo), and secured against off-study use (only one application per day possible). Home administration under the visual control of a physician (involving tDCS and its entire course) was allowed as an option for participants after thorough training (with online physician assistance and visual monitoring of tDCS engagement and progress) to reduce the burden of daily commuting to the NIMH. During each tDCS administration, patients performed activities (reading, housework, etc.) that they found difficult after the COVID-19 infection (not applied at rest to activate a dysfunctional cortical circuit).

### Data analysis

The baseline demographic and clinical characteristics between groups were compared using unpaired t-tests, Mann–Whitney tests, or Fisher's exact tests. Efficacy and safety data were obtained from the modified intention-to-treat (mITT) dataset, consisting of patients randomized to treatment and receiving at least one tDCS session. Data were treated as observed without imputation of missing data. To estimate changes in the primary outcome measurement, the FIS total score, from baseline to week four and follow-up, a mixed model for repeated measures (MMRM) with a restricted maximum likelihood (REML) approach and Kenward-Roger adjustment of degrees of freedom was employed. The model included fixed effects for the time, treatment, and their interaction, subjects treated as random effects, and baseline score, age, BMI, PASC duration, depression (PHQ-9), and anxiety (GAD 7) as covariates. The first-order autoregressive (AR1) correlation structure was used, but alternative structures were also assessed using the Akaike Information Criterion (AIC). Least-squares (LS) means, within and between-group differences in LS means, and corresponding 95% confidence intervals were calculated, and Sidak correction was applied. Secondary outcomes (FIS subscales, A-PASC total score and subscales, PHQ-9, GAD-7, cognitive tests, and AQOL-6D) were subjected to the same MMRM analysis, with covariate selection adapted to each outcome. The occurrence of side effects was compared by Fisher´s exact test. All statistical analyses were conducted using Stata Statistical Software 15 (StataCorp. 2017).

## Data Availability

After the publication of this article, de-anonymized data will be made available for non-commercial academic projects. Data can be obtained by request to the corresponding author. The de-anonymized data files with a dictionary will be provided via a secure data transfer service.
